# Glycoengineering of EphA4 Fc leads to a unique, long-acting and broad spectrum, Eph receptor therapeutic antagonist

**DOI:** 10.1038/s41598-017-06685-z

**Published:** 2017-07-26

**Authors:** Cassandra L. Pegg, Leanne T. Cooper, Jing Zhao, Michael Gerometta, Fiona M. Smith, Michael Yeh, Perry F. Bartlett, Jeffrey J. Gorman, Andrew W. Boyd

**Affiliations:** 1 Protein Discovery Centre, QIMR Berghofer Medical Research Institute, Queensland, 4006 Australia; 2 Leukaemia Foundation Research Laboratory, QIMR Berghofer Medical Research Institute, Queensland, 4006 Australia; 3 Queensland Brain Institute, University of Queensland, Queensland, 4072 Australia; 4 The Australian Institute for Bioengineering and Nanotechnology, University of Queensland, Queensland, 4006 Australia; 5 School of Chemistry and Molecular Biosciences, University of Queensland, Queensland, 4072 Australia; 6 Faculty of Medicine and Biomedical Sciences, University of Queensland, Queensland, 4006 Australia

## Abstract

Eph receptors have emerged as targets for therapy in both neoplastic and non-neoplastic disease, however, particularly in non-neoplastic diseases, redundancy of function limits the effectiveness of targeting individual Eph proteins. We have shown previously that a soluble fusion protein, where the EphA4 ectodomain was fused to IgG Fc (EphA4 Fc), was an effective therapy in acute injuries and demonstrated that EphA4 Fc was a broad spectrum Eph/ephrin antagonist. However, a very short *in vivo* half-life effectively limited its therapeutic development. We report a unique glycoengineering approach to enhance the half-life of EphA4 Fc. Progressive deletion of three demonstrated N-linked sites in EphA4 progressively increased *in vivo* half-life such that the triple mutant protein showed dramatically improved pharmacokinetic characteristics. Importantly, protein stability, affinity for ephrin ligands and antagonism of cell expressed EphA4 was fully preserved, enabling it to be developed as a broad spectrum Eph/ephrin antagonist for use in both acute and chronic diseases.

## Introduction

The Eph/ephrin system consists of the Eph receptor tyrosine kinases (RTK) and their ephrin ligands. The sixteen Eph RTK found in vertebrates are divided into A and B sub-groups based on structural features and their preference for A or B type ephrins respectively^1^. A striking feature of EphA4 is its ability to interact widely with both types of ephrin ligands, the structural basis of which has been extensively investigated^2–4^. This promiscuity has prompted interest in EphA4 as a number of Eph and ephrin proteins have been implicated in spinal cord injury and brain trauma^5–11^. Furthermore, expression of EphA4 has been shown to contribute to the pathology of disease and injury in the nervous system^6, 12^. It follows that blocking an individual Eph or ephrin protein might not constitute an effective therapy and that a broader blockade might be required. In this context, an EphA4 Fc soluble receptor decoy was shown to be effective in promoting recovery after spinal cord injury (SCI) where ephrin-B3 is the key ligand^6, 13^. Similarly, EphA4 Fc was shown to be highly effective in an ischemia-reperfusion model^14^ where EphA2 binding to ephrin-A1 is the key pathogenic mechanism. Whilst the use of EphA4 Fc as a “pan-ephrin blocker” has proven efficacy, our preliminary data suggested that it was relatively short-lived *in vivo* and thus unsuitable for use as a human therapeutic. Evidence that EphA4 plays a significant role in amyotrophic lateral sclerosis or motor neuron disease (MND)^12^, where prolonged therapy would be required, prompted us to explore the development of a more long-lived agent which could be delivered as a weekly bolus injection.

One way to enhance the pharmacokinetic properties of a therapeutic is to adopt a glycoengineering approach by modifying its carbohydrate content. Typically this is achieved by introducing N-linked consensus sites (Asparagine-X-Serine/Threonine, where X is not proline) into the protein sequence^15–20^ or by altering the protein glycosylation pattern^21^, thereby minimising receptor-mediated clearance through asialoglycoprotein and mannose receptors that recognise glycoproteins with specific glycan structures^22–24^. Glycoengineering does not usually involve removal of N-linked consensus sites as this has been found to result in reduced protein expression, a loss of function or increased serum clearance^25–27^. Contrastingly, we found that systematic mutation of three N-linked consensus sites in the EphA4 ectodomain, which were confirmed to contain glycans, improved pharmacokinetic profiles. Furthermore, the removal of all three sites did not interfere with binding affinities to key ephrin proteins or affect binding to cell surface ephrins. The form bearing all three mutations was shown to have a greatly extended half-life compared with glycosylated EphA4 Fc and to have pharmacokinetic properties compatible with its use as a weekly parenteral therapy.

## Results

### Protein sequence and glycosylation of native EphA4 Fc

**Figure 1:**
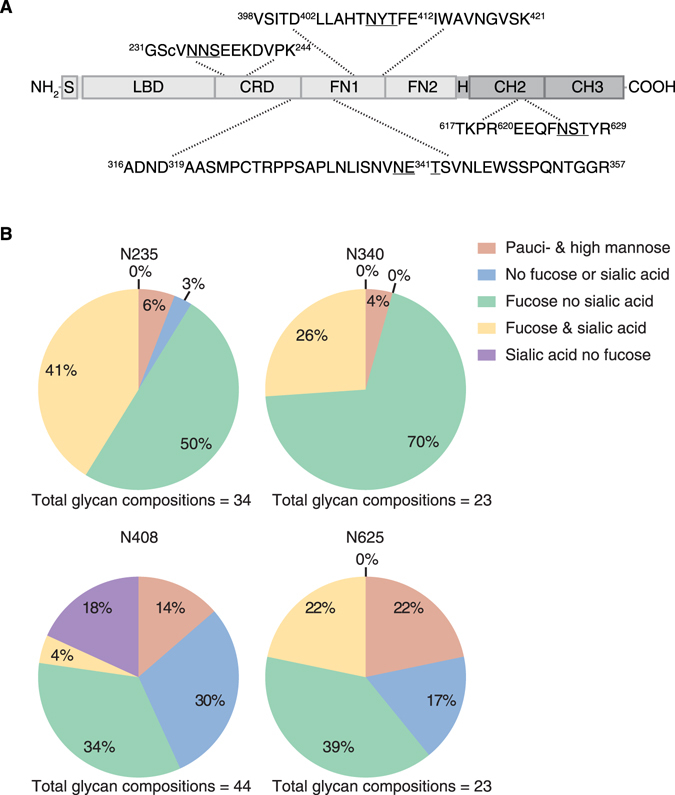
Glycosylation of wild type EphA4 Fc. (**A**) A schematic of EphA4 Fc (not to scale) identifying the EphA4 region (light grey) and the Fc region (dark grey). The EphA4 signal peptide (S) is located at the N-terminus of the protein and is followed by the ligand-binding domain (LBD), cysteine-rich domain (CRD) and two fibronectin type III repeats (FN1) and (FN2)^4^. The IgG4 Fc portion of the protein includes the hinge (H) region and the C_H_2 and C_H_3 constant immunoglobulin domains. Observed tryptic peptides containing the four N-linked sites (N235, N340, N408 and N625) are displayed with the N-linked consensus sites underlined. The amino- and carboxyl-terminal residues of the tryptic peptides have been annotated with the respective amino acid numbers from the EphA4 Fc sequence. Any additional observed proteolytic cleavage by trypsin or Glu-C within the tryptic peptide sequences have also been annotated with the respective amino acid numbers. (**B**) Qualitative distribution of the glycan compositions observed from EphA4 Fc at N-linked sites N235, N340, N408 and N625.

The EphA4 Fc fusion protein contains four predicted N-linked consensus sites (Fig. 1A); three are located in the EphA4 portion, corresponding to sites N235, N340 and N408 in the extracellular domain of full length EphA4 (UniProt ID: P54764–1). The fourth N-linked site, site N625, resides within the IgG4 Fc domain and corresponds to N297 in the C_H_2 domain of each heavy chain of all IgG subclasses^28^. The EphA4 Fc protein was digested with trypsin, which cleaves peptides bonds C-terminal to lysine (Lys/K) and arginine (Arg/R) except where proline resides on the carboxyl side of the cleavage site, before analysis by mass spectrometry (MS). EphA4 Fc was identified as the top scoring protein with 93% sequence coverage (Supplementary Fig. S1 and Table S1) when searched against a protein database containing the human proteome. The high level of sequence coverage indicated the correct protein was purified and confirmed the expected amino acid sequence. Peptides containing each of the four N-linked consensus sites from EphA4 Fc were observed and the peptide matches were manually validated.

To comprehensively investigate site-specific glycan heterogeneity of EphA4 Fc produced in HEK293T cells, an aliquot of the trypsin digested EphA4 Fc was also subjected to digestion with Glu-C, which cleaves peptide bonds C-terminal to glutamic acid (Glu/E) and at a lower rate C-terminal to aspartic acid (Asp/D)^29^. The trypsin and trypsin/Glu-C digested samples were analysed in separate chromatographic MS/MS experiments. The results confirmed occupancy at each of the four potential N-linked glycosylation sites with a total of 34, 23, 44 and 23 glycans detected at sites N235, N340, N408, and N625, respectively (Supplementary Tables S2 and S3). The qualitative difference of glycan compositions at each N-linked site revealed the proportion of glycans with sialylation (N-Acetylneuraminic acid/NeuAc) was highest at site N235 (41%) followed by sites N340 (26%), N408 (22%) and N625 (22%) (Fig. 1B). The relative abundances of glycopeptides containing each N-linked site were also assessed using extracted ion chromatograms^30^ (Supplementary Fig. S2), revealing that some of the most predominant glycoforms at N235 were sialylated.

### Identification of an EphA4 Fc candidate drug through site-directed mutagenesis

**Figure 2:**
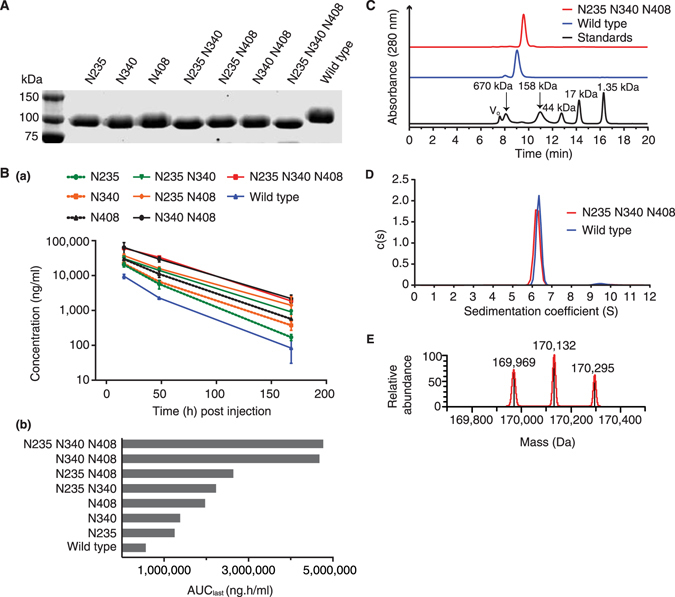
Identification of a mutagenic candidate of EphA4 Fc. (**A**) SDS-PAGE analysis of reduced, protein A purified single, double and triple mutants (N > Q) and wild type EphA4 Fc proteins. (**B**) Sandwich ELISA of single, double and triple mutants (N > Q) and wild type EphA4 Fc protein clearance in *Mus musculus* (a) with calculated AUC_last_ values (b). Protein conformation and stability of the triple mutant compared to wild type EphA4 Fc proteins using SEC (**C**) and SV-AUC (**D**). The profiles reveal single predominant peaks for the triple mutant and wild type EphA4 Fc proteins indicating the absence of significantly different sized protein species. The chromatogram for the molecular weight standards has been included in the SEC profile with the mass of the standards noted above the respective peak. **(E)** Deconvoluted MS spectrum of the intact triple mutant of EphA4 Fc showing the masses of the predominate species.

To analyse the effect of glycosylation on protein function we systematically mutated nucleotides in the human EphA4 Fc expression vector such that the codons for the three key asparagine (Asn/N) residues in the EphA4 extracellular domain (N235, N340 and N408) were replaced with codons for glutamine (Gln/Q) residues. The resulting single, double and triple mutant EphA4 Fc expression vectors were transfected into HEK293T cells and proteins were purified from cell supernatants using protein A affinity chromatography. All seven mutant proteins showed an increased rate of electrophoretic mobility compared to that of the “wild type” protein (Fig. 2A), consistent with glycosylation status. To assess if glycosylation was determining *in vivo* clearance we investigated the pharmacokinetic behaviour of the wild type EphA4 Fc protein and that of the single, double and triple mutant proteins in mice (Fig. 2B). All the mutations resulted in some prolongation of *in vivo* half-life but this was least pronounced in single mutants, more robust with double mutants and most prolonged when all three glycosylation sites were deleted. Values for the area under the plasma concentration-time curve from time zero to time of last measurable concentration (AUC_last_) ranged from 566,000 to 4,770,000 ng.h/ml (Fig. 2B). We then selected for further study the triple mutant of EphA4 Fc as it displayed a promising pharmacokinetic profile that may be compatible with therapeutic use and was produced as efficiently as the wild type protein (yields of ~0.2 g/L for both constructs). Based on these observations we also mutated the two predicted N-linked consensus sequences of EphA2 and three N-linked consensus sequences in the extracellular domain of EphB4. However, the half-life of the mutated EphA2 Fc protein was not increased compared to the wild type (Supplementary Fig. S3) and EphB4 was unsuccessful due to protein instability (data not shown).

### Structure and stability of the triple mutant of EphA4 Fc

Conformational differences and the oligomeric state of the un-mutated wild type and triple mutant EphA4 Fc proteins were assessed by size exclusion chromatography (SEC) (Fig. 2C) and sedimentation velocity analytical ultracentrifugation (SV-AUC) (Fig. 2D). During SEC there was a slight shift in the elution times with the triple mutant of EphA4 Fc eluting at 9.58 min while the wild type eluted at 9.03 min. The SV-AUC data revealed a narrow distribution of apparent sedimentation coefficients with predominant peaks at ~6.3 S for the wild type and ~6.2 S for the triple mutant. This confirmed the homogeneity observed in SDS-PAGE and SEC. The fitted frictional ratios indicated that the proteins have an elongated conformation and a slightly higher ratio was observed for the wild type protein compared to the triple mutant (1.76 versus 1.66). Molecular weight estimations of 174 kDa and 156 kDa were given for the wild type and triple mutant, respectively. These estimations are in agreement with those calculated for a homodimer of EphA4 Fc (~168 kDa), with the lower molecular weight of the triple mutant consistent with removal of the N-glycans. The mass estimation of the triple mutant protein was confirmed by intact MS (Fig. 2E) which revealed three predominant species with average masses around 170 kDa. The intact species were separated by ~163 Da and this observed heterogeneity is consistent with N-linked glycosylation of the remaining N-linked site (N625). At the glycopeptide level (Supplementary Fig. S2B) predominant glycoforms at site N625 were differentiated by hexose (162.05 Da) content.

### Ligand-binding activity and inhibition of EphA4 activation with the triple mutant of EphA4 Fc

**Figure 3:**
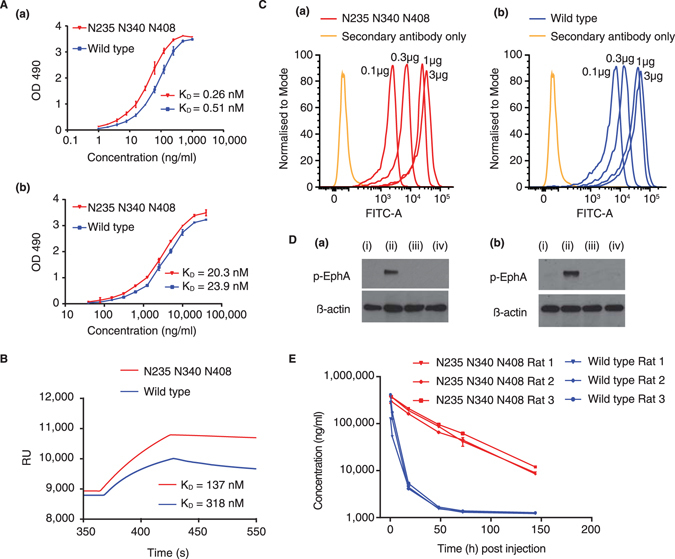
Ligand-binding and pharmacokinetic analyses of the triple mutant of EphA4 Fc compared to the wild type protein. (**A**) Ephrin-binding ELISA of the triple mutant and wild type EphA4 Fc proteins with immobilised ephrin-A5 Fc (a) and ephrin-B3 Fc (b). The apparent dissociation constants (K_D_) values are shown for the triple mutant and wild type proteins. (**B**) Kinetic analyses of the interactions of the wild type and triple mutant EphA4 Fc proteins were determined by surface plasmon resonance measurements using a BIAcore biosensor with sensorchip surfaces immobilised with ephrin-A5 Fc. (**C**) Flow cytometric analysis of binding of the triple mutant (a) and wild type (b) EphA4 Fc proteins to ephrin-A5 expressing CHO cell line. (**D**) Inhibition of receptor activation using EphA4 stably transfected CHO cell line and pre-clustered (a) ephrin-A5 Fc (b) ephrin-B3 Fc with (i) unstimulated (ii) pre-clustered ephrin (iii) pre-clustered ephrin with wildtype EphA4 and (iv) pre-clustered ephrin with mutant EphA4. (**E**) Sandwich ELISA of the triple mutant and wild type EphA4 Fc protein clearance in *Rattus norvegicus*.

Analysis of ligand binding of the triple mutant and un-mutated wild type EphA4 Fc proteins was carried out using several methods: ephrin-binding ELISA (Fig. 3A), surface plasmon resonance (SPR) (Fig. 3B), and flow cytometric analysis of binding to ephrin-expressing cell lines (Fig. 3C). These techniques revealed that the triple mutant retained the ability to bind to ephrin ligands with a comparable affinity to the wild type EphA4 Fc protein. Apparent affinity measurements obtained through ELISA for the wild type and triple mutant (Fig. 3A) were within reported ranges for EphA4 binding to ephrin-A5 and ephrin-B3 using similar methods^31^. Likewise, affinity measurements obtained from SPR for the wild type and triple mutant binding to ephrin-A5 (Fig. 3B) were similar to those reported in the literature^4^ (values for k_a1_ [M^−1^s^−1^], k_d1_[s^−1^], k_a2_ [RU^−1^s^−1^] and k_d2_ [s^−1^] were 5.63 × 10^4^, 2.24 × 10^−2^, 1.12 × 10^−4^ and 1.06 × 10^−3^ for the wild type and 1.15 × 10^5^, 1.84 × 10^−2^, 5.40 × 10^−5^ and 2.66 × 10^−6^ for the mutant, respectively^32^). The affinity data shows the triple mutant has slightly stronger affinity for the ephrin ligands although values all fell within the nanomolar range. Taken together, the data implied that a concentration of >1 µg/ml of the triple mutant would saturate all ephrin-A5 ligands and that >10 µg/ml would be sufficient to saturate ephrin-B3 sites. We also confirmed that both the wild type and triple mutant proteins similarly blocked both clustered ephrin-A5 and clustered ephrin-B3-induced EphA4 phosphorylation in Chinese Hamster Ovary (CHO) cells stably transfected with EphA4 (Fig. 3D).

### Pharmacokinetic analysis of the triple mutant of EphA4 Fc

When the pharmacokinetics of the triple mutant was compared to the un-mutated wild type protein directly in rats (performed in triplicate) a more dramatic prolongation of half-life of the triple mutant was observed, whereas the un-mutated protein was cleared very rapidly (Fig. 3E). It is evident that the triple mutant protein exhibits a simple log-linear clearance in contrast to the un-mutated protein which shows a more complex rapid initial clearance followed by a plateau, consistent with the heterogeneity of glycosylation at the predicted Asn residues. The C_max_ (maximum serum concentration) values, corresponding to the initial sample taken at 15 min for each protein, were similar at 318,000 ng/ml and 343,000 ng/ml for the wild type and triple mutant respectively. However, the AUC_last_ values were 1,620,000 ng.h/ml and 12,700,000 ng.h/ml for the wild type and triple mutant respectively, an increase of 7.8 fold for the latter. The elimination half-life for the triple mutant was estimated at 31.1 h while the wild type half-life could not be estimated due to the plateau reached at 48 h.

## Discussion

The Eph/ephrin receptor ligand system constitutes an important signalling pathway and a growing number of studies have revealed the efficacy of ephrin and Eph fusion proteins in ameliorating several disease models^33, 34^. Notably, EphA4 exhibits promiscuous binding affinities and has been identified as a promising target for MND and SCI^6, 12, 13^. Currently, treatments for such diseases and injuries are limited and therapeutic candidates such as EphA4 Fc may fulfil these substantial unmet medical needs. In preliminary studies we found that EphA4 Fc was cleared rapidly, requiring frequent dosing in mouse and rat SCI studies^35^. As glycosylation has previously been shown to affect the pharmacokinetic properties of therapeutic proteins^36^ we implemented a glycoengineering approach to enhance the half-life of EphA4 Fc, thus potentially enabling reduced frequency of administration.

It has been demonstrated that *in vivo* clearance of Fc-fusion proteins can be dependent on the structure of N-linked glycans^21, 37^. To explore the possible involvement of glycosylation in rapid clearance of EphA4 Fc we investigated the glycosylation profile of the protein. Mass spectral analysis of glycopeptides derived from EphA4 Fc confirmed that a number of different glycan compositions, including high mannose and hybrid or complex-types, were attached at each of the four predicted N-linked sites. Consistent with prediction, no O-linked glycosylation sites within the EphA4 region of the protein were detected. It has been previously demonstrated that the distribution of glycans in the Fc domain of Fc-fusion proteins remains unchanged *in vivo* ^37^, likely due to its relative sequestration in the C_H_2–C_H_3 domain, making interaction with glycan-binding receptors sterically unfavoured. We therefore sought to determine if sites in the EphA4 domain directly impacted pharmacokinetic properties. Systematic mutation of N-linked consensus sites, individually and in combination, revealed varying levels of prolonged half-life compared to the wild type protein (Fig. 2B). Interestingly, mutation of site N235 had the least impact on clearance which is consistent with the observation of a higher proportion of sialylated glycans attached at this site (Fig. 1B and Supplementary Fig. S2A). Mechanistically, the higher degree of sialylation at site N235 may reduce the level of asialoglycoprotein or mannose receptor-mediated clearance, thus accounting for the minimal impact of mutating this site on clearance^38^. It should be noted that the pattern of glycan compositions and the pharmacokinetic profile of wild type EphA4 Fc are specific to EphA4 Fc expressed in HEK293T cells and may not reflect the profiles of endogenously produced EphA4 or those expressed in other cell lines. Furthermore, endogenous EphA4 may exhibit more complete glycosylation compared to wild type EphA4(-Fc) overexpressed in HEK293T, where sialylation is relatively low.

Whilst the mutation of individual sites showed a variable effect, the protein with a completely un-glycosylated EphA4 region showed the greatest prolongation of availability. These results are unusual in the context of glycoengineering, where studies have demonstrated that increasing the number of glycosylation sites improves pharmacokinetic profiles^15–20^ while removing sites increases clearance^27^. An exception to this was the removal of one N-linked site in tissue plasminogen activator which contained high mannose glycans^39^. Notably, the inability to achieve the same result with EphA2 in this work suggests that the mutation strategy cannot be generalised to all Eph proteins. The rapid clearance of both wild type and mutated EphA2 Fc suggests clearance was not driven by glycosylation characteristics. Other mechanisms for rapid clearance of antibodies and Fc fusion proteins may include off-target and target-mediated binding^40^.

The removal of glycans may also be detrimental for protein integrity as glycans often serve to stabilise proteins and prevent proteolysis and aggregation. This was illustrated in the production of non-glycosylated ephrin-A1 which resulted in misfolding of the protein^25^. This effect has also been observed in other proteins where mutating N-glycosylation sites resulted in significant losses of protein expression^41, 42^. However, we demonstrated that the structure and stability of the triple mutant of EphA4 Fc was maintained. To investiagte if this was a common characteristic of all Eph proteins, consensus sites for N-glycans from EphB4 were also mutated but the resulting mutant was not stable. The purity, conformation and oligomeric state of the triple mutant of EphA4 Fc were compared to wild type using SDS-PAGE, SEC and SV-AUC (Fig. 2A,C and D, respectively). As judged by SV-AUC both the triple mutant and wild type EphA4 Fc appear to be stable dimers with molecular weights in reasonably close agreement to those calculated, when taking into account N-glycan differences. Interestingly, the SEC elution times of the mutant and wild type (9.58 and 9.03 min, respectively) are significantly higher than the expected elution based on theoretical molecular weight, which should correspond to bovine γ-globin (158 kDa, elution time of 11.0 min), suggesting both molecules have a non-globular structure. This conforms to observations from crystal structures of the ectodomain of EphA4, which reveal that the proteins form oblong or ridged “rod-like” structures^3, 43^. This may have implications for the function of membrane bound Eph receptors where an extended rod-like structure of the extracellular domain may facilitate interaction with the relatively small ephrin ligands. Taken together, the results of SEC and SV-AUC indicate that wild type EphA4 has a slightly larger hydrodynamic volume or more elongated conformation (consistent with earlier elution in SEC and a higher fitted frictional ratio in SV-AUC). Thus, in the context of EphA4 Fc, glycosylation may contribute to the slightly higher degree of rigidity seen in the wild type protein compared to the mutant.

Altering glycosylation can also affect protein function, for example, deglycosylation of ephrin-A1 had a negative impact on its binding affinity with EphA2^25^, while the introduction of certain N-linked sites into recombinant human erythropoietin reduced *in vitro* activity^17^. It is therefore significant that the triple mutant of EphA4 Fc retained *in vitro* binding activity with ephrin ligands ephrin-A5 and ephrin-B3 (Fig. 3A–C). The triple mutant of EphA4 Fc also inhibited clustered ephrin-A5 and ephrin-B3 EphA4 phosphorylation in CHO cells in a similar manner to the wild type protein (Fig. 3D). These are important binding interactions for EphA4 and have been highlighted in disease settings^6, 7, 35^. These results provide an insight into the role of N-linked glycosylation in EphA4, revealing that N-linked consensus sites are not essential for ligand binding properties. Such information is particularly useful as the batch-to-batch consistency of un-glycosylated therapeutics are less likely to be affected by variability in glycosylation^44^.

Pharmacokinetic analyses revealed that wild type EphA4 Fc exhibited rapid clearance in the first 24–48 h, accounting for the vast majority of circulating drug, but then plateaued for the remaining time points (Fig. 3E). This may be due to preferred clearance of EphA4 Fc glycoforms containing asialylated N-glycans, while those glycoforms containing sialylated glycans or without N-linked glycosylation were retained *in vivo*. The pharmacokinetic studies show a pronounced effect of glycosylation on the protein systemic exposure half-life *in vivo*, where there was a 7.8 fold improvement in the AUC_last_. It is notable that the terminal half-life of the triple mutant of 31.1 h may have been underestimated in this preliminary experiment, as the full terminal elimination phase was unlikely to have been captured in the data set. Importantly, in the rat experiment a dose of 6 mg/kg was able to sustain a serum concentration which, based on a concentration of 10 µg/ml being able to bind maximally to all ephrin-A and ephrin-B ligands, would block all ephrin sites for 6–7 days. This is highly significant as it provides the potential to enable dosing with EphA4 Fc at weekly intervals as opposed to the glycosylated protein which would need to be delivered daily or by continuous infusion to be effective as a decoy receptor drug.

The ability of EphA4 to effectively bind ephrin-A and -B ligands makes it an ideal therapeutic candidate and offers a significant advantage over small molecules that selectively inhibit specific Ephs or ephrins. Using a comprehensive set of assays profiling structural, functional and pharmacokinetic properties, we have provided a unique approach to extend the half-life of a broad spectrum Eph receptor antagonist. Although the data is limited to *in vitro* and animal models it supports the advancement of EphA4 Fc for clinical applications. This development of a long acting antagonist therefore removes a major obstacle in the development of a pharmacological inhibitor or Eph/ephrin interactions for use in both acute and chronic diseases.

## Methods

### Site directed mutagenesis

Nucleotides coding for Asn at predicted glycosylation sites 235, 340 and 408 of EphA4 Fc and sites N407 and N435 of EphA2 Fc were mutated sequentially to encode glutamine using Quik Change Site Directed Mutagenesis (Agilent Technologies Inc., Santa Clara, CA). Plasmid DNA was purified using a QIAGEN plasmid miniprep kit prior to confirmation of mutagenesis of glycosylation sites by DNA sequencing.

### Transient transfections of plasmid DNA

HEK293T cells (ATCC, CRL-3216) were transfected with plasmid DNA using Lipofectamine 2000 (Invitrogen) according to manufacturer’s guidelines. Culture supernatant was harvested on day 6 post transfection and purified by Protein A affinity chromatography using MabSelect agarose matrix (GE Healthcare Life Sciences).

### SDS-PAGE analysis

Protein A purified EphA4 Fc samples were subjected to SDS-PAGE analysis in the presence of the reducing agent β mercaptoethanol. Briefly, samples were electrophoresed on a 10% (v/v) SDS polyacrylamide gel followed by staining with Coomassie Brilliant Blue R-250 (Sigma).

### Binding ELISA assay

EIA 96 well plates (Costar, Corning Inc.) were coated with ephrin-A5 Fc or ephrin-B3 Fc proteins (3 µg/ml) in 50 mM carbonate buffer pH 9.5 overnight at 4 °C prior to blocking with 5% (w/v) BSA, 0.05% (v/v) Tween-20 in PBS (PBST) for 1 h at room temperature. Following three washes with PBST, diluted EphA4 Fc analyte was added, incubated for 1 h room temperature, washed three times with PBST prior to incubation with biotinylated 1A7mAb (1 µg/ml) in PBS for 1 h room temperature. After three washes with PBST, Ultra Streptavidin-HRP (Thermo Fischer Scientific) diluted 1/500 in PBS was added, incubated 1 h at room temperature and washed three times with PBST. Final detection was via the addition of OPD (Sigma*Fast ™*) with the end product measured at OD 492 nm. The Kd values were calculated by nonlinear regression using GraphPad Prism (v7.0) and the “One site – Specific binding” model.

### FACS analysis

The CHO ephrin-A5 expressing cell line was incubated with either the EphA4 Fc triple mutant or wild type proteins (0.1 to 3 µg/ml) for 1 h at 4 °C, washed twice 5% (v/v) FCS in PBS prior to staining for 30 min at 4 °C with conjugated Alexa Fluor 488 goat anti-human IgG (Technologies, Carlsbad, CA, USA) conjugated secondary antibody at 1:400 dilution. Following three washes in 5% (v/v) FCS in PBS cells were analysed on BD LSR Fortessa flow cytometer using FACS DIVA software.

### Surface Plasmon Resonance

Analysis of protein interactions by surface plasmon resonance was carried out on a BIAcore T200 biosensor (GE Healthcare). Ephrin-A5 Fc was immobilised on CM5 sensorchip (GE Healthcare, BR100530) via standard amine coupling procedure (GE Healthcare, BR100050). A blank immobilisation was used as control. Binding of the triple mutant of EphA4 Fc and wild type EphA4 Fc proteins, diluted between 500 and 50 nM into running buffer (10 mM HEPES, pH 7.4, 150 mM NaCl, 3 mM EDTA, 0.05% v/v Surfactant P20), was performed on ephrin-A5 Fc derived flow cell and blank flow cell simultaneously. The kinetic experiments were carried out at 30 µl/min and the surface of the chip was regenerated with a 30 second injection of 3 M MgCl_2_, followed by a 60 second wait. The interaction kinetics was derived from the plasmon resonance sensorgrams after subtraction of baseline responses (measured on the control flow cell) by global analysis using the BIAcore T200 Evaluation Software (version 1.0, GE Healthcare). Bivalent analyte model and steady state affinity model were applied for fitting of the sensorgram data.

### EphA4 receptor signalling assay

Both ephrin-A5 Fc and ephrin-B3 Fc (20 µg/ml) were pre-clustered with anti-human-IgG (10 µg/ml) (Sigma Aldrich) at a 2:1 ratio in serum free media for 1 h at 4 °C. Mutant or wildtype EphA4-Fc at 20 µg/ml was added to CHO cells stably expressing EphA4, prior to the addition of the pre-clustered ephrins at 1 µg/ml. After 30 min (37 °C), cells were washed twice with ice cold PBS, then lysed with buffer containing 1% Triton X-100, 1 mM EDTA, 150 mM NaCl, 20 mM TrisHCl pH7.4, with the addition of cOmplete protease inhibitor cocktail (Roche) and PhosSTOP phosphatase inhibitor tablets (Roche). Proteins were separated using SDS-PAGE, Western blotted with protein detected using anti-Eph receptor A2 + A3 + A4 polyclonal antibody (Abcam), and beta actin polyclonal antibody (CST), followed by either anti-rabbit-HRP conjugated or anti-mouse-HRP conjugated secondary antibody for chemiluminescence detection.

### Pharmacokinetic analysis of EphA4 Fc (*Mus musculus*)

Twenty-four C57BL/6 mice (8 weeks of age) were randomly divided into eight groups (three per group), with the mutants of EphA4 Fc or wild type proteins at 5 mg/kg administered by intraperitoneal injection. Serum was collected at 18 h, 48 h, and 7 days post injection by retro-orbital bleeds and collection of 150–200 µl of blood into a Microvette blood collection tube. Following incubation for 30–45 min in an upright position at room temperature, tubes were centrifuged at 11,000 rpm for 10 min prior to serum removal. Serum was stored at −80 °C prior to ELISA analysis. Pharmacokinetic parameters for all analyses were estimated by non-compartmental methods using Phoenix 64 WinNonlin® software (Certara USA, Inc., Princeton, NJ). All animal experiments were conducted in accordance with the Australian Code of Practice for the Care and Use of Animals for Scientific Purposes, including housing of animals and procedural guidelines. Animal breeding and experimental ethical approval was obtained from the University of Queensland Animal Ethics Committee.

### Pharmacokinetic analysis of the triple mutant of EphA4 Fc (*Rattus norvegicus*)

Six Wistar rats (8 weeks of age) were randomly divided into two groups (three per group) and injected with either the triple mutant of EphA4 Fc or wild type proteins at 6 mg/kg through tail vein injections. Serum was collected at 0.25 h, 2 h, 18 h, 48 h, 72 h and 6 days post injection by cutting the tips of tails and collection of 150–200 µl of blood into a Microvette blood collection tube. Serum was isolated as previously described and stored at −80 °C prior to ELISA analysis.

### Pharmacokinetic analysis of EphA2 Fc (*Rattus norvegicus*)

Similarly, six Wistar rats at age of 8 weeks (three per group) were intravenously injected with either the double mutant of EphA2 Fc or wild type proteins at 1 mg/kg through tail vein injections, with blood collection at 2 h, 18 h, 48 h, and 6 days. The remaining procedures for sample collection, processing and storage, were as previously described.

### EphA4 Fc Sandwich ELISA assay

EIA 96 well plates (Costar, Corning Inc.) were coated with 1F9 (anti-EphA4) mAb (3 µg/ml) in 50 mM carbonate buffer pH 9.5 overnight at 4 °C prior to blocking with 5% (w/v) BSA, 0.05% (v/v) Tween-20 in PBS (PBST) for 1 h at room temperature. Following three washes with PBST, diluted EphA4Fc analyte was added, incubated for 1 h at room temperature, washed three times with PBST prior to incubation with biotinylated 1A7(anti-EphA4)mAb (1 µg/ml) in PBS for 1 h at room temperature. After three washes with PBST, Ultra Streptavidin-HRP (Thermo Fischer Scientific) diluted 1/500 in PBS was added, incubated 1 h at room temperature and washed three times with PBST. Final detection was via the addition of OPD (Sigma*Fast™*) with the end product measured at OD 492 nm.

### EphA2 Fc Sandwich ELISA assay

The EphA2 sandwich was essentially as described above, except the anti-EphA2 4B3 coating antibody and anti EphA2 biotinylated 1F7 antibody were used for detection.

### Enzymatic digestion of EphA4 Fc

Production of peptides and glycopeptides from wild type EphA4 Fc was accomplished by reducing 115 µg of protein with 10 mM dithiothreitol in 100 mM of NH_4_HCO_3_ buffer with 1% (w/v) SDS at 4 °C for 18 h. This was followed by further reduction for 2 h at room temperature before cysteine residues were alkylated using 25 mM iodoacetamide for 2 h in the dark. The reduced and alkylated proteins were methanol-precipitated with trypsin (Roche Diagnostics GmbH, Mannheim, Germany) as previously described^45^ with a final ratio of protein: enzyme, 38:1 (w/w). An aliquot of the trypsin digested protein was further digested with Glu-C isolated from *Staphylococcus aureus* V8 (Roche Diagnostics GmbH, Mannheim, Germany) at 37 °C for 12 h (protein: enzyme, 40:1, w/w). After Glu-C digestion the resultant peptides and glycopeptides were desalted with a C18 ZipTip (10 µl pipette tip with a 0.6 µl resin bed; Millipore, MA, USA) using the manufactures’ guidelines before MS analysis.

### LC-MS/MS analysis

Mass spectral analysis was performed on an Orbitrap Fusion™ Tribrid™ Mass Spectrometer (Thermo Fischer Scientific, San Jose, CA) coupled to a nanoACQUITY UPLC (nUHPLC) system (Waters Corporation, MA), where trapping was performed on a Waters C18 2 G Symmetry (100 Å, 5 µm, 180 µm × 20 mm) trap column and gradient elution on a Waters C18 BEH (130 Å, 1.7 µm particle size, 75 µm × 200 mm) column in-line with the trap column. Digested samples were acidified with trifluoroacetic acid before 50 ng of the wild type EphA4 Fc tryptic digest and 100 ng of the trypsin/Glu-C digest were injected for each analysis. Samples were loaded onto the trap and washed over 5 min using 98% solvent A (0.1% (v/v) aqueous formic acid) and 2% solvent B (100% (v/v) CH_3_CN containing 0.1% (v/v) formic acid) at 15 µl/min. Peptides were subsequently eluted onto the analytical column at flow rate of 0.3 µl/min whilst ramping through a sequence of linear gradients from 2% to 40% solvent B in 60 min, to 70% B over 15 min, to 95% B in 5 min and holding at 95% B for 5 min. Eluates from the analytical column were continuously introduced into the mass spectrometers via a Nanospray Flex™ (NG) ion Source (Thermo Scientific) fitted with a PicoTip™ emitter (coating P200P, tip 10 ± 1 μm, New Objective, MA).

MS survey scans of nUHPLC-fractionated tryptic peptides was performed in the Orbitrap over the range of 300–1800 (m/z) at a resolution of 120 K at 200 m/z with an automatic gain control (AGC) target of 200,000 and a maximum injection time of 50 ms. The most intense precursors with charge states of 2–7 and intensities greater than 5,000 were selected using an isolation window of 2.5 and fragmented by higher-energy (beam-type) C-trap dissociation using 30% normalised collision energy. Mass analysis of the fragment ions was performed in the Orbitrap where the resolution was set at 60 K with an AGC target of 50,000 and a maximum injection time of 60 ms. Once selected the precursors were excluded for 30 sec.

### Data analysis of non-glycosylated peptides

The MS RAW file generated from the analysis of trypsin digested EphA4 Fc was searched using Proteome Discoverer (v. 1.4.1.14 Thermo Fisher Scientific Inc. Bremen, Germany) and the search engine Mascot (v. 2.5.1, Matrix Science Ltd., London, UK) with the Percolator function against a complete human proteome database (ID: UP000005640 with 70,076 sequences downloaded from www.uniprot.org on 13 January 2016) and the EphA4 Fc sequence. The following parameters were used: digestion with trypsin; maximum two missed cleavages; 10 ppm precursor mass tolerance; 0.02 Da fragment tolerance; fixed modification of carbamidomethyl cysteine and dynamic modifications of mono-oxidised methionine and deamidation of Asn and Gln. Confident peptide-to-spectrum matches were assigned using a false discovery rate threshold of 0.05 and two distinct peptides were required for confident protein identifications.

### Data analysis of glycosylated peptides

The MS RAW data files from the trypsin and trypsin/Glu-C digestions of EphA4 Fc were analysed with Byonic software^46^ using the same mass tolerances and fixed modifications from the Proteome Discoverer search. The enzyme rules were set as cleavage C-terminal to Arg or Lys (trypsin digested sample) or C-terminal to Arg, Lys, Glu or Asp (combined trypsin/Glu-C digestion) and missed cleavages were set to two for the trypsin digested sample and three for the trypsin/Glu-C sample. One O-linked or N-linked glycan and one mono-oxidised methionine were allowed per peptide. The peptide output options were changed to “Show all N-glycopeptides” and the spectra were searched against a protein database containing the sequence for EphA4 Fc and a Byonic O-linked glycan (six common O-linked) and N-linked (309_Mammalian no sodium) database with all glycans compositions containing N-glycolylneuraminic acid removed. After manual inspection of the Byonic results a cut-off score of 100 was chosen for glycopeptides.

The MS RAW files from the trypsin and trypsin/Glu-C digestions of EphA4 Fc were additionally converted to mzML in Proteome Discoverer using a signal to noise threshold of zero. The files were analysed with OxoExtract^47^, an in-house software program that searched selected glycopeptide spectra in GlycoMod^48^ to determine the composition of the attached N-linked glycan. This data was used to verify glycopeptides assigned by Byonic, confirm the presence of relevant oxonium and glycopeptide ions and search for potential glycopeptides not assigned by Byonic.

The minimum requirement to accept any glycopeptide assignment was the presence of the peptide + N-acetylhexosamine ion or greater than four peptide b- and y-ions. If the sequence of the peptide moiety included a methionine residue at least two peptide b- or y-ions were required to evidence potential oxidation or lack thereof. Relevant glycan oxonium ions had to be present to validate the assigned glycan composition and the elution profile was checked to ensure glycopeptides were eluting at expected retention times. Once a list of allocated glycopeptides had been complied the Xtract feature within Xcalibur Qual Browser was used to deconvolute eluting glycopeptide precursor ions from MS spectra for annotation of glycopeptides. Precursor MS spectra were summed across the elution period for all glycoforms containing the relevant peptide sequence. Monoisotopic masses were generated as [M] using a signal to noise threshold of two and a maximum charge state of five.

### Size exclusion chromatography

The triple mutant and wild type EphA4 Fc proteins were injected onto a TSK-GEL SWXL guard column (6.0 mm ID × 400 mm, 7-μm particles) and subsequently eluted onto a TSK-GEL G3000SWXL HPLC column (7.8 mm ID × 300 mm, 5-μm particles) using an Agilent 1200 chromatography system. The flow rate was 0.8 ml/min and the mobile phase contained 100 mM sodium phosphate (pH 6.8) and 200 mM NaCl. Eluted proteins were monitored by UV absorption (280 nm wavelength). The following standards were used: thyroglobin (670 kDa), bovine γ-globin (158 kDa), ovalbumin (44 kDa), equine myoglobin (17 kDa), and vitamin B_12_ (1.35 kDa) (BioRad Laboratories, Hercules, CA, USA).

### Analytical ultracentrifugation

The triple mutant and wild type EphA4 Fc proteins were dialysed overnight against a buffer containing 10 mM potassium phosphate (pH 7.6). Samples were analysed using an XL-I analytical ultracentrifuge (Beckman Coulter, Fullerton, CA) equipped with an AnTi-60 rotor. Protein samples were loaded in the sample compartment of double-sector epon centrepieces, with buffer in the reference compartment. Radial absorbance data was acquired at 20 °C using a rotor speed of 40,000 rpm and a wavelength of 280 nm, with radial increments of 0.003 cm in continuous scanning mode. The sedimenting boundaries were fitted to a model that describes the sedimentation of a distribution of sedimentation coefficients with no assumption of heterogeneity (c(s)) using the program SEDFIT^49^. Data were fitted using a regularisation parameter of p = 0.95, floating frictional ratios, and 150 sedimentation coefficient increments in the range of 0.1–15 S.

### Intact protein mass analysis

For intact MS, the triple mutant of EphA4 Fc was exchanged into PBS and approximately 10 μg was injected onto a C4 PepMap300 Precolumn (5 μm 300 Å, particle size, 300 μm ID × 5 mm) and separated on an Acclaim PepMap 300 column (75 μm ID × 150 mm) at a flow rate of 300 nl/min. A gradient of 10–98% buffer B over 15 min was used, where solvent A was 0.1% (v/v) aqueous formic acid and solvent B was 99.9% (v/v) CH_3_CN containing 0.1% (v/v) formic acid. Proteins eluted from the column were directly analysed on an Orbitrap ELITE (Thermo Fisher Scientific Inc. Bremen, Germany) mass spectrometer interfaced with a NanoFlex source. The instrument was operated in positive ion mode using the ion trap for mass analysis. Source parameters included an ion spray voltage of 2 kV, temperature at 275 °C, SID = 70 V, S-lens = 70 V. MS analysis was performed across a mass range of 1000–3000 m/z. Data was deconvoluted using Thermo Scientific™ Protein Deconvolution software across a retention time of 12–14 min, and 1200–2200 m/z.

## Electronic supplementary material

Supplementary Information

Supplementary Table S1

Supplementary Table S2

Supplementary Table S3

## References

[1] Lemke G (1997). A coherent nomenclature for Eph receptors and their ligands. Mol. Cell. Neurosci..

[2] Singla N (2010). Crystal structure of the ligand-binding domain of the promiscuous EphA4 receptor reveals two distinct conformations. Biochem. Biophys. Res. Commun..

[3] Xu K (2013). Insights into Eph receptor tyrosine kinase activation from crystal structures of the EphA4 ectodomain and its complex with ephrin-A5. Proc. Natl. Acad. Sci. USA.

[4] Bowden TA (2009). Structural plasticity of eph receptor A4 facilitates cross-class ephrin signaling. Structure.

[5] Arocho LC (2011). Expression profile and role of EphrinA1 ligand after spinal cord injury. Cell. Mol. Neurobiol..

[6] Goldshmit Y, Galea MP, Wise G, Bartlett PF, Turnley AM (2004). Axonal regeneration and lack of astrocytic gliosis in EphA4-deficient mice. J. Neurosci..

[7] Qu Y, Zhao J, Wang Y, Gao Z (2014). Silencing ephrinB3 improves functional recovery following spinal cord injury. Mol. Med. Rep..

[8] Fabes J (2006). Accumulation of the inhibitory receptor EphA4 may prevent regeneration of corticospinal tract axons following lesion. Eur. J. Neurosci..

[9] Miranda JD (1999). Induction of Eph B3 after spinal cord injury. Exp Neurol..

[10] Willson CA, Miranda JD, Foster RD, Onifer SM, Whittemore SR (2003). Transection of the adult rat spinal cord upregulates EphB3 receptor and ligand expression. Cell Transplant..

[11] Ujigo S (2014). Administration of microRNA-210 promotes spinal cord regeneration in mice. Spine (Phila Pa 1976).

[12] Van Hoecke A (2012). EPHA4 is a disease modifier of amyotrophic lateral sclerosis in animal models and in humans. Nat. Med..

[13] Spanevello MD (2013). Acute delivery of EphA4-Fc improves functional recovery after contusive spinal cord injury in rats. J. Neurotrauma.

[14] Woodruff TM (2016). Epha4-Fc treatment reduces ischemia/reperfusion-induced intestinal injury by inhibiting vascular permeability. Shock.

[15] Egrie JC, Browne JK (2001). Development and characterization of novel erythropoiesis stimulating protein (NESP). Nephrol. Dial. Transplant..

[16] Egrie JC, Dwyer E, Browne JK, Hitz A, Lykos MA (2003). Darbepoetin alfa has a longer circulating half-life and greater *in vivo* potency than recombinant human erythropoietin. Exp. Hematol..

[17] Elliott S (2003). Enhancement of therapeutic protein *in vivo* activities through glycoengineering. Nat. Biotechnol..

[18] Perlman S (2003). Glycosylation of an N-terminal extension prolongs the half-life and increases the *in vivo* activity of follicle stimulating hormone. J. Clin. Endocrinol. Metab..

[19] Ceaglio N, Etcheverrigaray M, Kratje R, Oggero M (2008). Novel long-lasting interferon alpha derivatives designed by glycoengineering. Biochimie.

[20] Stork R (2008). N-glycosylation as novel strategy to improve pharmacokinetic properties of bispecific single-chain diabodies. J. Biol. Chem..

[21] Liu L (2013). The impact of glycosylation on the pharmacokinetics of a TNFR2:Fc fusion protein expressed in Glycoengineered Pichia Pastoris. Pharm. Res..

[22] Ashwell G, Harford J (1982). Carbohydrate-specific receptors of the liver. Annu. Rev. Biochem..

[23] Stahl PD (1992). The mannose receptor and other macrophage lectins. Curr. Opin. Immunol..

[24] Stockert RJ (1995). The asialoglycoprotein receptor: relationships between structure, function, and expression. Physiol. Rev..

[25] Ferluga S (2013). Biological and structural characterization of glycosylation on ephrin-A1, a preferred ligand for EphA2 receptor tyrosine kinase. J. Biol. Chem..

[26] Tsai IH, Wang YM, Huang KF (2015). Effects of single N-glycosylation site knockout on folding and defibrinogenating activities of acutobin recombinants from HEK293T. Toxicon.

[27] Ni H, Blajchman MA, Ananthanarayanan VS, Smith IJ, Sheffield WP (2000). Mutation of any site of N-Linked glycosylation accelerates the *in vivo* clearance of recombinant rabbit antithrombin. Thromb. Res..

[28] Zauner G (2013). Glycoproteomic analysis of antibodies. Mol. Cell. Proteomics.

[29] Breddam K, Meldal M (1992). Substrate preferences of glutamic-acid-specific endopeptidases assessed by synthetic peptide substrates based on intramolecular fluorescence quenching. Eur. J. Biochem..

[30] Yang Y (2016). Hybrid mass spectrometry approaches in glycoprotein analysis and their usage in scoring biosimilarity. Nat. Commun..

[31] Noberini R, Rubio de la Torre E, Pasquale EB (2012). Profiling Eph receptor expression in cells and tissues: A targeted mass spectrometry approach. Cell Adhesion & Migration.

[32] Pabbisetty KB (2007). Kinetic analysis of the binding of monomeric and dimeric ephrins to Eph receptors: Correlation to function in a growth cone collapse assay. Protein Sci..

[33] Boyd AW, Bartlett PF, Lackmann M (2014). Therapeutic targeting of EPH receptors and their ligands. Nat. Rev. Drug Discov..

[34] Barquilla A, Pasquale EB (2015). Eph receptors and ephrins: therapeutic opportunities. Annu. Rev. Pharmacol. Toxicol..

[35] Goldshmit Y (2011). EphA4 blockers promote axonal regeneration and functional recovery following spinal cord injury in mice. PLoS One.

[36] Higel F, Seidl A, Sorgel F, Friess W (2016). N-glycosylation heterogeneity and the influence on structure, function and pharmacokinetics of monoclonal antibodies and Fc fusion proteins. Eur. J. Pharm. Biopharm..

[37] Jones AJS (2007). Selective clearance of glycoforms of a complex glycoprotein pharmaceutical caused by terminal N-acetylglucosamine is similar in humans and cynomolgus monkeys. Glycobiology.

[38] Liu L (2015). Antibody glycosylation and its impact on the pharmacokinetics and pharmacodynamics of monoclonal antibodies and Fc-fusion proteins. J. Pharm. Sci..

[39] Cole ES, Nichols EH, Poisson L, Harnois ML, Livingston DJ (1993). *In vivo* clearance of tissue plasminogen activator: The complex role of sites of glycosylation and level of sialylation. Fibrinolysis.

[40] Tabrizi MA, Tseng CM, Roskos LK (2006). Elimination mechanisms of therapeutic monoclonal antibodies. Drug Discov Today.

[41] Takahashi M (2008). N-glycan of ErbB family plays a crucial role in dimer formation and tumor promotion. Biochim. Biophys. Acta.

[42] Mishina M (1985). Location of functional regions of acetylcholine receptor alpha-subunit by site-directed mutagenesis. Nature.

[43] Seiradake E (2013). Structurally encoded intraclass differences in EphA clusters drive distinct cell responses. Nat. Struct. Mol. Biol..

[44] Schiestl M (2011). Acceptable changes in quality attributes of glycosylated biopharmaceuticals. Nat. Biotechnol..

[45] Dave KA, Headlam MJ, Wallis TP, Gorman J (2011). J. Preparation and analysis of proteins and peptides using MALDI TOF/TOF mass spectrometry. Curr. Protoc. Protein Sci..

[46] Bern, M., Kil, Y. J. & Becker, C. Byonic: Advanced Peptide and Protein Identification Software. *Curr. Protoc. Bioinformatics* **13.20**, 13.20.11-13.20.14, (2012). 10.1002/0471250953.bi1320s40 PMC3545648 23255153

[47] Pegg CL, Hoogland C, Gorman JJ (2017). Site-specific glycosylation of the Newcastle disease virus haemagglutinin-neuraminidase. Glycoconj. J..

[48] Cooper CA, Gasteiger E, Packer NH (2001). GlycoMod – A software tool for determining glycosylation compositions from mass spectrometric data. Proteomics.

[49] Schuck P, Rossmanith P (2000). Determination of the sedimentation coefficient distribution by least-squares boundary modeling. Biopolymers.

