# Supplementary material for: Glycoengineering of EphA4 Fc leads to a unique, long-acting and broad spectrum, Eph receptor therapeutic antagonist
Source: Sci Rep. 2017 Jul 26;7:6519. doi: 10.1038/s41598-017-06685-z (PMC5529513; doi:10.1038/s41598-017-06685-z)
Supplement: Supplementary file 1 — Supplementary Information [file 41598_2017_6685_MOESM1_ESM.pdf]

From: Glycoengineering of EphA4 Fc leads to a unique, long-acting and broad spectrum, Eph receptor therapeutic antagonist

Cassandra L. Pegg, Leanne T. Cooper, Jing Zhao, Michael Gerometta, Fiona M. Smith, Michael Yeh, Perry F. Bartlett, Jeffrey J. Gorman and Andrew W. Boyd

```
1    MAGIFYFALF SCLFGICDAV TGSRVYPANE VTLLDSRSVQ GELGWIASPL EGGWEEVSIM DEKNTPIRTY QVCNVMEPSQ
81   NNWLRTDWIT REGAQRVYIE IKFTLRDCNS LPGVMGTCKE TFNLYYYESD NDKERFIREN QFVKIDTIAA DESFTQVDIG
161  DRIMKLNTEI RDVGPLSKKG FYLAFQDVGA CIALVSVRVF YKKCPLTVRN LAQFPDTITG ADTSSLVEVR GSCVNNSEEK
241  DVPKMYCGAD GEWLVPIGNC LCNAGHEERS GECQACKIGY YKALSTDATC AKCPPHSYSV WEGATSCTCD RGFFRADNDA
321  ASMPCTRPPS APLNLISNVN ETSVNLEWSS PQNTGGRQDI SYNVVCKKCG AGDPSKCRPC GSGVHYTPQQ NGLKTTKVSI
401  TDLLAHTNYT FEIWAVNGVS KYNPNPDQSV SVTVTTNQAA PSSIALVQAK EVTRYSVALA WLEPDRPNGV ILEYEVKYYE
481  KDQNERSYRI VRTAARNTDI KGLNPLTSYV FHVRARTAAG YGDFSEPLEV TTNTVPSRII GDGANSESKY GPPCPPCPAP
561  EFLGGPSVFL FPPKPKDTLM ISRTPEVTCV VVDVSQEDPE VQFNWYVDGV EVHNAKTKPR EEQFNSTYRV VSVLTVLHQD
641  WLNGKEYKCK VSNKGLPSSI EKTISKAKGQ PREPQVYTLP PSQEEMTKNQ VSLTCLVKGF YPSDIAVEWE SNGQPENNYK
721  TTPPVLDSDG SFFLYSRLTV DKSRWQEGNV FSCSVMHEAL HNHYTQKSLS LSLGK
```

Supplementary Figure S1. Sequence coverage of EphA4 Fc. Protein sequence coverage of EphA4 Fc based on identified tryptic peptides by HCD MS/MS. Sequence coverage derived from the Mascot search has been highlighted in dark grey.

a

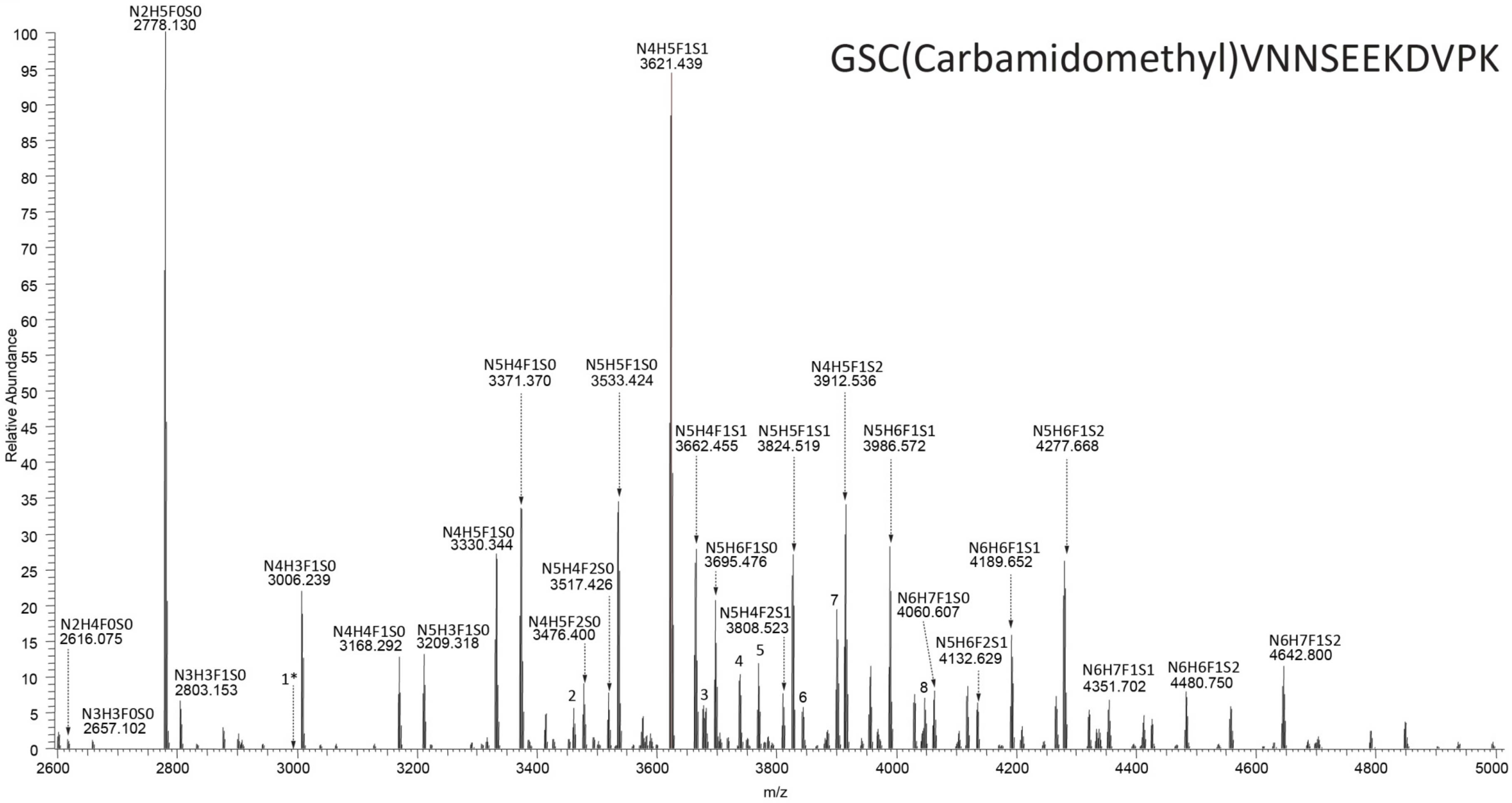
GSC(Carbamidomethyl)VNNSEEKDVPK
Relative Abundance
m/z
N2H5F0S0 2778.130
N4H5F1S1 3621.439
N5H4F1S0 3371.370
N5H5F1S0 3533.424
N4H5F1S2 3912.536
N5H4F1S1 3662.455
N5H5F1S1 3824.519
N5H6F1S1 3986.572
N5H6F1S2 4277.668
N4H5F1S0 3330.344
N5H6F1S0 3695.476
N6H6F1S1 4189.652
N5H4F2S0 3517.426
N4H3F1S0 3006.239
N6H7F1S0 4060.607
N5H4F2S1 3808.523
N4H5F2S0 3476.400
N2H4F0S0 2616.075
N4H4F1S0 3168.292
N5H3F1S0 3209.318
N5H6F2S1 4132.629
N6H7F1S2 4642.800
N3H3F1S0 2803.153
N6H7F1S1 4351.702
N6H6F1S2 4480.750
N3H3F0S0 2657.102
1*
2
3
4
5
6
7
8

b

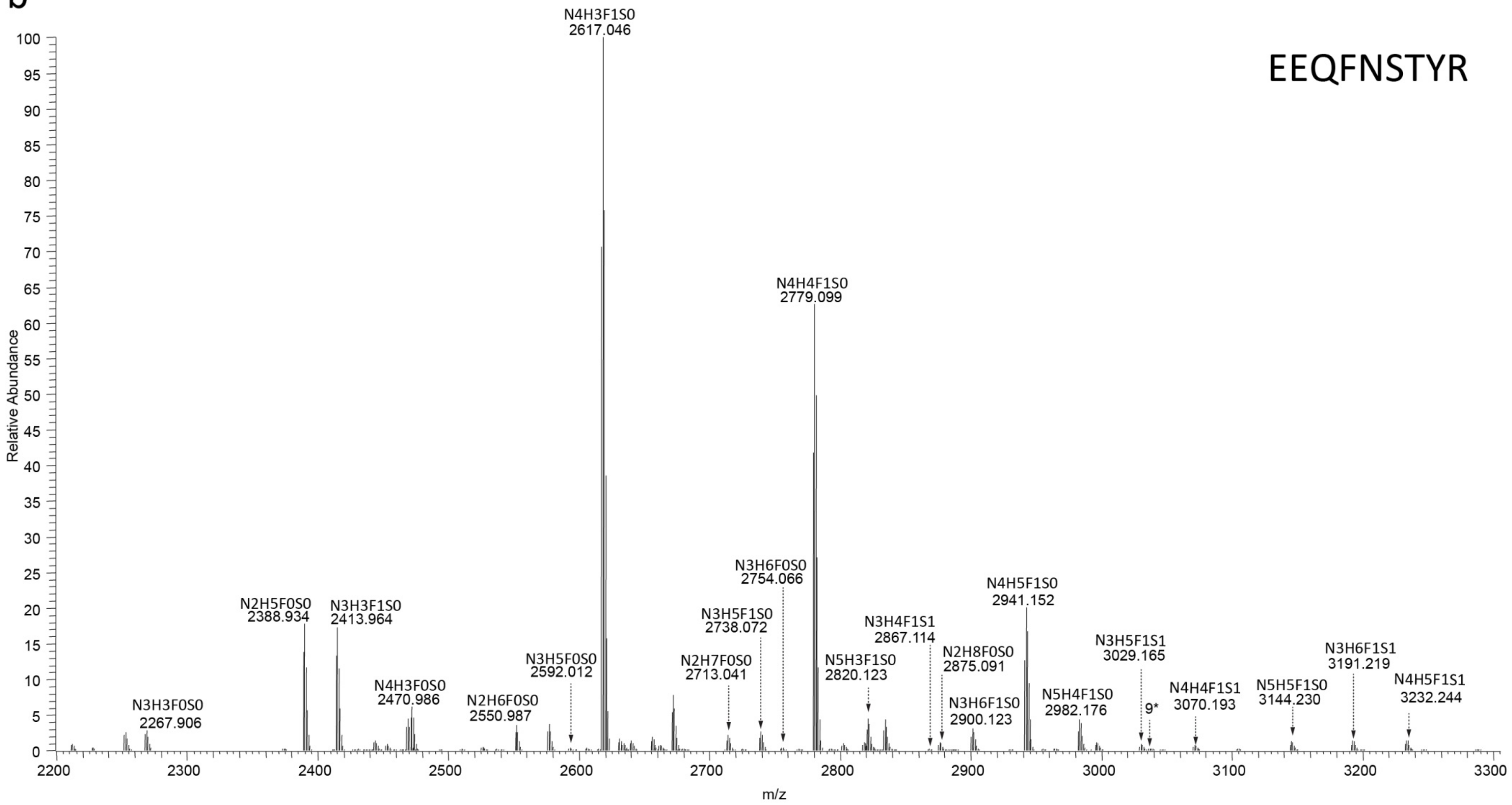
EEQFNSTYR
Relative Abundance
m/z
N4H3F1S0
2617.046
N4H4F1S0
2779.099
N3H3F0S0
2267.906
N2H5F0S0
2388.934
N3H3F1S0
2413.964
N4H3F0S0
2470.986
N2H6F0S0
2550.987
N3H5F0S0
2592.012
N2H7F0S0
2713.041
N3H5F1S0
2738.072
N3H6F0S0
2754.066
N5H3F1S0
2820.123
N3H4F1S1
2867.114
N2H8F0S0
2875.091
N3H6F1S0
2900.123
N4H5F1S0
2941.152
N5H4F1S0
2982.176
N3H5F1S1
3029.165
9*
N4H4F1S1
3070.193
N5H5F1S0
3144.230
N3H6F1S1
3191.219
N4H5F1S1
3232.244

c

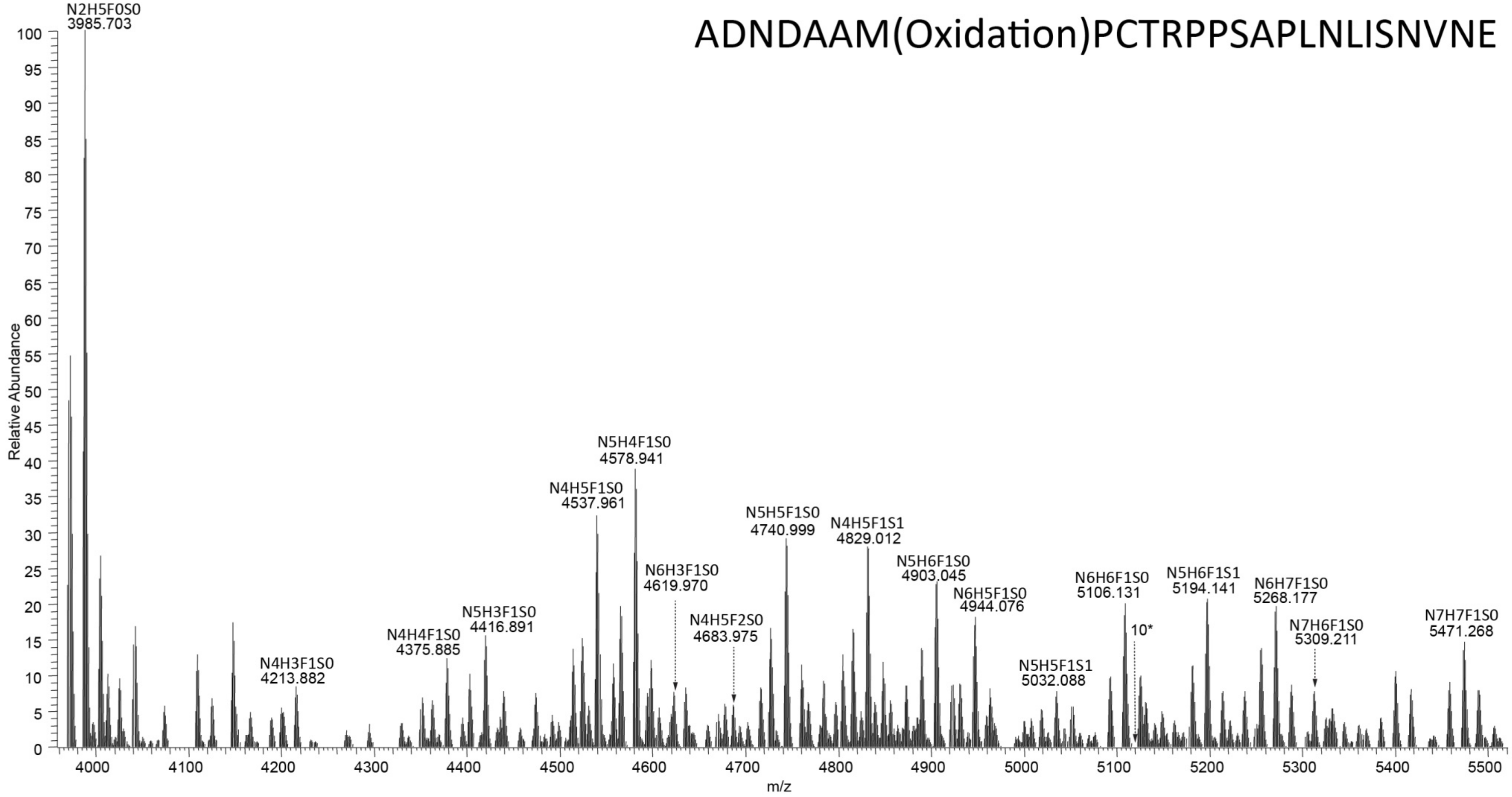
ADNDAAM(Oxidation)PCTRPPSAPLNLISNVNE
N2H5F0S0
3985.703
N4H3F1S0
4213.882
N4H4F1S0
4375.885
N5H3F1S0
4416.891
N4H5F1S0
4537.961
N5H4F1S0
4578.941
N6H3F1S0
4619.970
N4H5F2S0
4683.975
N5H5F1S0
4740.999
N4H5F1S1
4829.012
N5H6F1S0
4903.045
N6H5F1S0
4944.076
N5H5F1S1
5032.088
N6H6F1S0
5106.131
10*
N5H6F1S1
5194.141
N6H7F1S0
5268.177
N7H6F1S0
5309.211
N7H7F1S0
5471.268
Relative Abundance
m/z

d

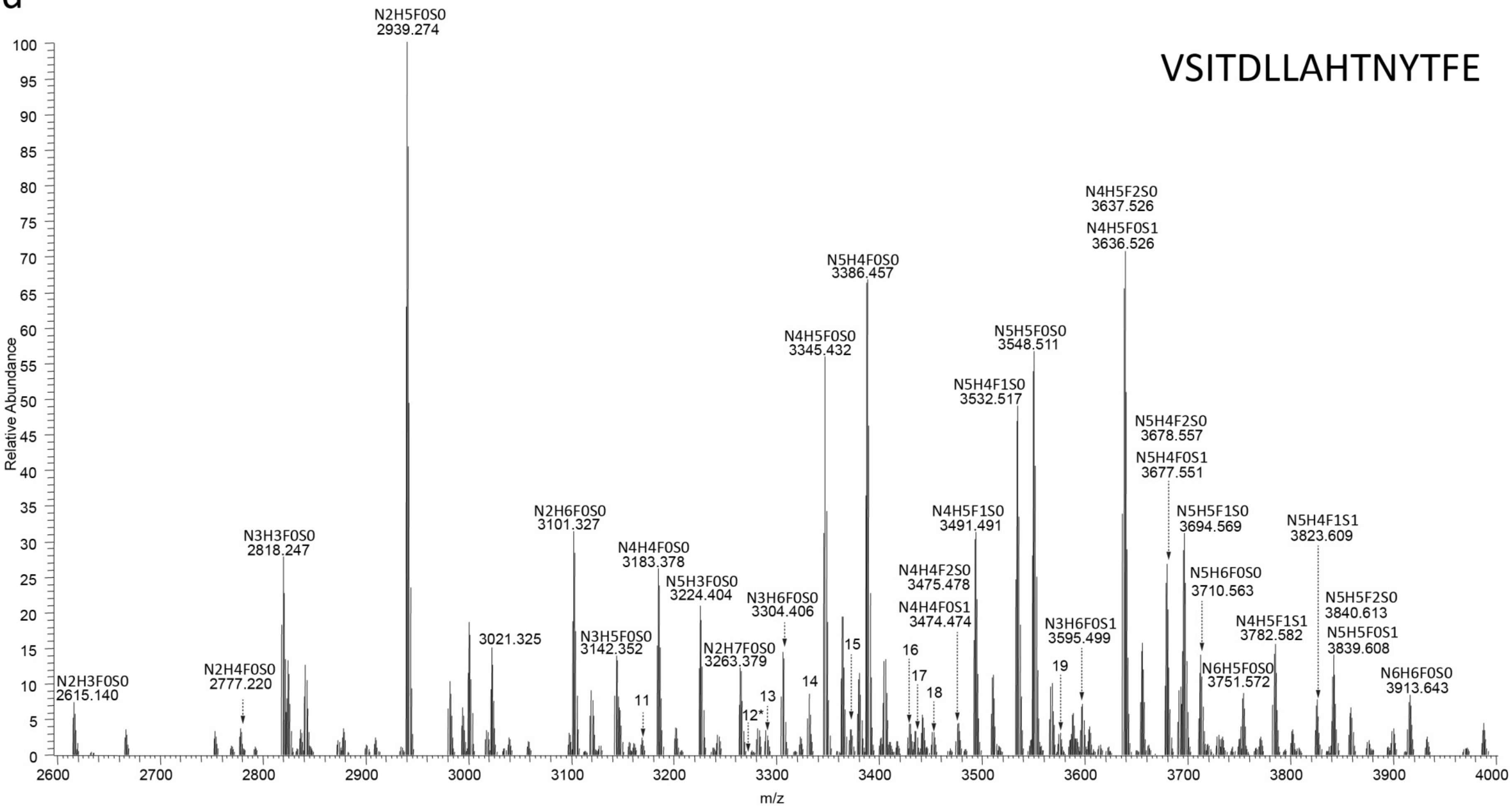
VSITDLLAHTNYTFE
N2H5F0S0
2939.274
N2H3F0S0
2615.140
N2H4F0S0
2777.220
N3H3F0S0
2818.247
3021.325
N2H6F0S0
3101.327
N3H5F0S0
3142.352
N4H4F0S0
3183.378
N5H3F0S0
3224.404
N2H7F0S0
3263.379
N3H6F0S0
3304.406
N4H5F0S0
3345.432
N5H4F0S0
3386.457
N4H4F2S0
3475.478
N4H4F0S1
3474.474
N4H5F1S0
3491.491
N5H4F1S0
3532.517
N5H5F0S0
3548.511
N3H6F0S1
3595.499
N4H5F2S0
3637.526
N4H5F0S1
3636.526
N5H4F2S0
3678.557
N5H4F0S1
3677.551
N5H5F1S0
3694.569
N5H6F0S0
3710.563
N6H5F0S0
3751.572
N4H5F1S1
3782.582
N5H4F1S1
3823.609
N5H5F2S0
3840.613
N5H5F0S1
3839.608
N6H6F0S0
3913.643
11
12*
13
14
15
16
17
18
19
Relative Abundance
m/z

Supplementary Figure S2. Deconvoluted spectra identifying glycopeptides containing each N-linked site from wildtype EphA4 Fc.

Glycan heterogeneity observed on EphA4 Fc trypsin digested peptides GSC(Carbamidomethyl)VNNSEEKDVPK (Panel. a) and EEQFNSTYR (Panel. b) and trypsin/Glu-C digested peptides ADNDAASM(oxidation)PCTRPPSAPLNLISNVNE (Panel. c) and VSITDLLAHTNYTFE (Panel. d) representing N-linked sites N235, N625, N340 and N408, respectively. MS precursor spectra were summed across the elution period for all glycoforms and deconvoluted from multiply charged signals to unprotonated monoisotopic masses using the Xtract feature within Xcalibur Qual Browser. Major signals representing glycopeptide precursors have been labelled with glycan compositions and precursor masses in the relevant sections of each spectrum. N= N-Acetylhexosamine, H= Hexose, F= Fucose (Deoxyhexose), S= Sialic acid (N-Acetylneuraminic acid). For ease of interpretation some of the minor signals have been labelled with numbers representing the corresponding glycan compositions: 1. N3H4F1S0; 2.N4H4F1S1; 3.N5H5F2S0; 4.N6H5F1S0; 5.N4H5F2S1; 6.N5H6F2S0; 7.N6H6F1S0; 8. N6H6F2S0; 9. N2H9F0S0; 10. N4H5F1S2; 11.N4H3F1S0; 12.N3H4F0S1; 13.N3H5F1S0; 14.N4H4F1S0; 15.N5H3F1S0; 16.N6H3F0S0; 17.N3H5F0S1; 18.N3H6F1S0; 19.N6H3F1S0. Numbers with an asterisk "*" denote precursors that were not present in the deconvoluted spectra. Please note that in one panel (Panel. d) four signals (~ m/z 3475, 3637, 3678 and 3840) have each been assigned two glycan compositions. The glycan compositions at each signal have the same number of HexNAc and Hex residues but one composition includes two fucose resides and the second a sialic acid residue. This occurs because the mass difference between two fucose (Fuc) resides and one sialic acid residue is 1.020 Da while the mass difference between ^{13}C and ^{12}C isotopic peaks is 1.003 Da, thus when summing and deconvoluting spectra the isotopic profiles overlap. Each composition was previously assigned based on retention time, precursor mass and the presence of relevant oxonium ions for Fuc and Sialic acid in the MS/MS spectra.

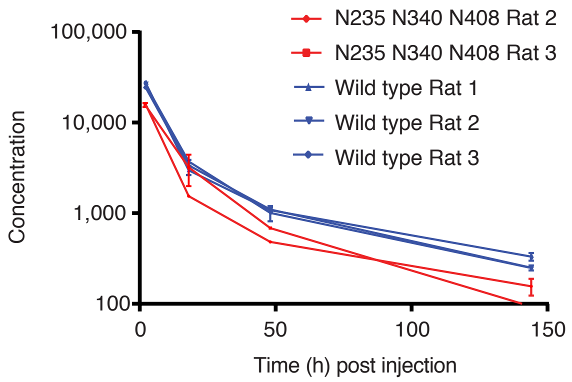

Supplementary Figure S3. Pharmacokinetic analysis of double mutant and wildtype EphA2 Fc proteins. Sandwich ELISA of the dual mutated EphA2 Fc (N407 and N435) and un-mutated EphA2 Fc (wild type) protein clearance in *Rattus norvegicus*. The numbering of asparagine residues that were mutated to glutamine corresponds to that of the amino acid sequence of human Ephrin type-A receptor 2 (UniProt identifier P29317). Data could not be obtained for Rat 1 injected with the double mutant of EphA2 Fc. The AUC_{last} was 431,000 ng.h/ml and 261,000 ng.h/ml for the wild type and triple mutant, respectively.
